# Changes in Cytochrome-C-Oxidase Account for Changes in Attenuation of Near-Infrared Light in the Healthy Infant Brain

**DOI:** 10.1007/978-3-319-91287-5_2

**Published:** 2018-01-01

**Authors:** M. F. Siddiqui, S. Lloyd-Fox, P. Kaynezhad, I. Tachtsidis, M. H. Johnson, C. E. Elwell

**Affiliations:** Centre for Brain and Cognitive Development, Department of Psychology, Birkbeck College, University of London, London, UK; Department of Medical Physics and Biomedical Engineering, University College London, London, UK; Centre for Brain and Cognitive Development, Department of Psychology, Birkbeck College, University of London, London, UK; Department of Medical Physics and Biomedical Engineering, University College London, London, UK

## Abstract

A novel multi-wavelength broadband near infrared spectroscopy (NIRS) system has been employed to simultaneously measure haemodynamic changes alongside changes in cellular oxygen utilization by measurement of oxidation state of mitochondrial enzyme cytochrome-c-oxidase (oxCCO). The aim of this study was to investigate the role of oxCCO in neural responses to functional activation in infants. Studies were performed using a NIRS broadband system in 33 typically developing infants aged between 4 and 6 months. Responses were recorded over the right temporal lobe while infants were presented with engaging videos containing social and nonsocial content. Changes in the concentration of oxyhaemoglobin (Δ[HbO_2_]), deoxyhaemoglobin (Δ[HHb]) and Δ[oxCCO] were calculated using changes in attenuation of light at 120 wavelengths between 780 and 900 nm using the UCLn algorithm. The algorithm was also used to fit (a) HbO_2_ and HHb spectra (2 component fit) and (b) HbO_2_, HHb and oxCCO (3 component fit) to the change in attenuation occurring within an experimental block in different participants. Residuals resulting from these two fits were compared with oxidized-minus reduced CCO spectrum, calculated using the CCO specific extinction coefficient. A significant increase in oxCCO was found in response to the social stimuli (maximum increase 0.238 ± 0.13 μM). Residuals analysis showed that the best fits were achieved when oxCCO was included as a tissue chromophore. These results are the first reported significant change in oxCCO to stimulus-evoked activation in infants and may reveal vital information about oxygen metabolism during functional activation in the developing human brain.

## 1 Introduction

NIRS is a non-invasive optical technique that provides valuable measures of cerebral oxygenation and haemodynamic changes through quantification of changes in oxygenated and deoxygenated haemoglobin Δ[HbO_2_] and Δ[HHb], by absorption of near-infrared light by underlying brain tissue. Over recent years, NIRS has become an established research tool for infant brain imaging in the field of developmental neuroscience and psychology and is being used to investigate both typical [1] and atypical development [2].

NIRS measures of cerebral haemodynamic changes provide useful information about oxygen delivery in the brain. However, oxygen delivery is only one component of the neurovascular coupling pathway, and there is a need to develop further measures of the supply/demand balance during functional activation. In particular, from a neurodevelopmental perspective, the haemodynamic response in infants can, in some circumstances, be difficult to interpret, is not yet fully understood, and its susceptibility to variation between infants makes it challenging to interpret and understand [3, 4]. Furthermore, HbO_2_ and HHb may be prone to physiological noise from systemic changes [5]. Recent technological advances in NIRS have allowed the measurement of cellular energy metabolism through measurement of mitochondrial respiratory chain enzyme cytochrome-c-oxidase (CCO). CCO is the terminal electron acceptor in the electron transport chain and is responsible for over 95% of oxygen metabolism in the body. The copper A redox centre of CCO, in its oxidized form, has a distinct absorption peak in the NIR spectrum. The total concentration of CCO in healthy individuals remains constant, therefore the NIRS measurement provides a marker of the oxidation state of CCO (oxCCO). Compared to haemoglobin based measures, oxCCO can potentially provide a more direct marker of brain activation, and animal studies [6] have found a significant correlation between oxCCO measures and phosphorus magnetic resonance spectroscopy biomarkers of cerebral energy metabolism.

However, due to the concentration of CCO in the brain being much lower than the concentrations of oxy- and deoxy-haemoglobin, the measurement of changes in CCO can be complicated and challenging. This might lead to the possibility that the measured Δ[oxCCO] could be the result of cross talk.

In this study, we used a broadband NIRS system to measure changes in oxCCO alongside haemodynamic changes in typical human infants, in response to functional activation. We analysed the residual errors produced when converting the attenuation of light into chromophore concentration changes to determine whether Δ[oxCCO] are accounted for in the attenuation change spectrum.

## 2 Materials and Methods

The study protocol and procedures were approved by the Birkbeck Psychology Research Ethics Committee. Thirty-three healthy 4-to-6-month-old infants participated in the study (14 males, 19 females, age 159 ± 25 days old). All parents volunteered and gave written, informed consent to participate.

### 2.1 Instrumentation

**Fig. 1:**
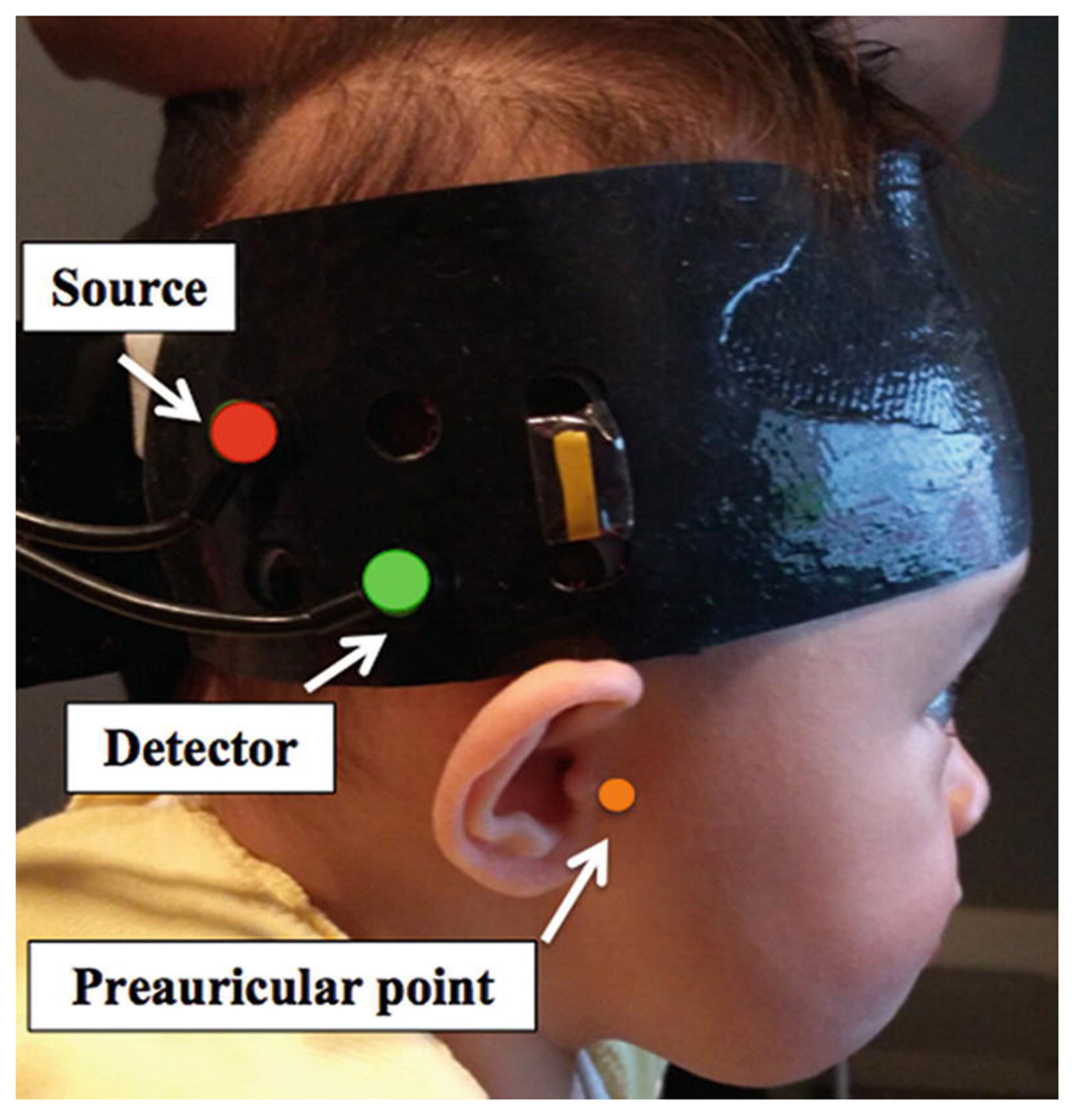
Positioning of the NIRS headgear on a participant’s head

Measurements were performed using a miniature broadband system, referred to as the mini-CYRIL [7], modified from a larger system (the Cytochrome Research Instrument and application system (CYRIL)) [8]. The system consisted of a miniature Ocean Optics HL2000 white light source using 20 W halogen-tungsten lamp and an Ocean Optics Ventana VIS-NIR spectrometer. The attenuation signal was obtained from changes in attenuation of light at 120 wavelengths between 780 and 900 nm and the sampling frequency was 1 Hz. mini-CYRIL consisted of a single channel and the infants wore custom-built 3D printed NIRS headgear containing a single source-detector pair with separation of 2.8 cm. A NIRS-MRI co-registration map [9] was used to place the single channel over the right superior temporal sulcus-temporo-parietal region, a brain area that has previously shown activation in infants of this age to social stimuli [10]. Figure 1 shows the placement of the array on an infant’s head.

### 2.2 Protocol and Measurements

Infants were seated on their parent’s lap during the study, approximately 1 m from a 46-in plasma screen which was used to display the stimuli. The experimental condition consisted of a visual and auditory component. The visual component consisted of dynamic social videos displaying biological motion, for example actors performing “incy-wincy”. The auditory component consisted of human vocals sounds, such as yawning. The baseline condition consisted of static transport images, for example cars and helicopters. Data was collected in a single session and both experimental and baseline conditions (9–12 s each) were alternated for a pseudorandom duration until the infants became bored or fussy. The average number of trials per infant was 13.

### 2.3 Data Analysis

Data analysis was carried out in MATLAB (Mathworks, USA). A detailed description of the data analysis pipeline is described elsewhere [11]. Valid experimental trials were determined using looking time. A trial was defined as valid if the infant looked at the screen for a minimum of 4 s prior to stimulus onset and looked at a minimum of 60% of the experimental condition. An infant was included in the study if they had a minimum of six valid trials and a typical haemodynamic response (specified as an increase in Δ[HbO_2_] and either a decrease or no change in Δ[HHb]) was exhibited in response to the stimulus. Past infant studies have reported the occurrence of an “inverted” haemodynamic response to the stimuli in infants, i.e. there is an increase in Δ[HHb] and a decrease in Δ[HbO_2_]. The mechanism that drives this response is unclear and is currently the subject of investigation by researchers. For this first study of cytochrome, we decided to exclude infants showing an inverted response however, in future studies we aim to use measures of cytochrome to investigate the processes driving the differing haeomdynamic responses during infancy.

A wavelet-based motion correction algorithm was applied to the attenuation signal of each subject with tuning parameter α = 1.5. Following this, the UCLn algorithm [12] was used to convert the attenuation change signal into Δ[HbO_2_], Δ[HHb] and Δ[oxCCO]. The wavelength-dependent differential pathlength factor used was 5.13 [13]. A 5th order Butterworth low pass filter with cut-off frequency of 0.225 Hz was used to filter the concentration changes. The data were epoched to obtain blocks which consisted of 4 s of the baseline condition preceding the experimental condition, the experimental condition as well as the entire succeeding baseline condition. Following this, the mean time courses of Δ[HbO_2_], Δ[HHb] and Δ[oxCCO] were obtained by averaging the valid blocks for each participant. These time courses were then combined to obtain a grand averaged concentration change time course for each of the chromophores, across all infants.

The UCLn algorithm was also used to perform the residual analysis. This involved using the algorithm to back-calculate the attenuation change spectra for each of the chromophores from the calculated concentration changes and then comparing this to the measured attenuation change occurring within an experimental block in different participants. First, only HbO_2_ and HHb attenuation change spectra were calculated (2-component fit) and then, HbO_2_, HHb and oxCCO attenuation change spectra were calculated (3-component fit) and compared to the measured attenuation change. Following this, the oxidized-minus reduced CCO spectrum was also calculated using the relevant specific extinction coefficient.

## 3 Results

Based on the exclusion criteria previously described, we included data from 24 out of 33 infants. Of the excluded infants, three were excluded for failing to look at the stimuli for the required minimum number of trials, one infant was excluded due to incorrect placement of the headgear on the infant’s head and five infants were excluded due to the absence of a typical haemodynamic response to functional activation as defined previously.

**Fig. 2:**
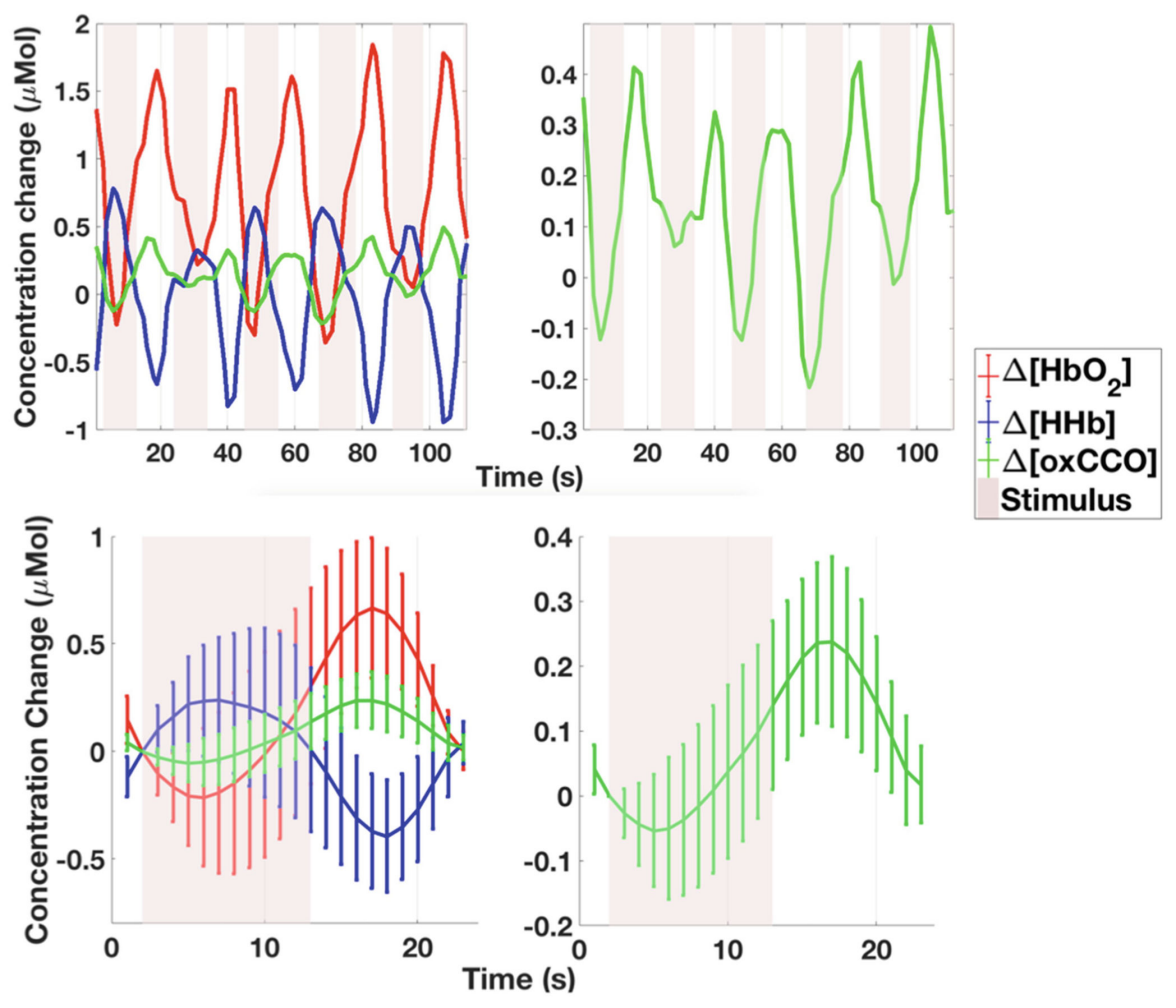
(**a**) Δ[HbO_2_], Δ[HHb] and Δ[oxCCO] from a single participant (**b**) Δ[oxCCO] with axis rescaled. (**c**) Grand averaged Δ[HbO_2_], Δ[HHb] and Δ[oxCCO] (**d**) Δ[oxCCO] with axis rescaled. The error bars represent standard deviation

Figure 2a presents Δ[HbO_2_], Δ[HHb] and Δ[oxCCO] time series from a single participant across five experimental trials, before averaging across trials. Figure 2b shows the same data with Δ[oxCCO] presented on a different scale.

Figure 2c displays the grand averaged chromophore concentration changes (averaged across 24 infants, across all valid experimental trials) and Fig. 2d shows grand averaged Δ[oxCCO] presented on a different scale.

**Fig. 3:**
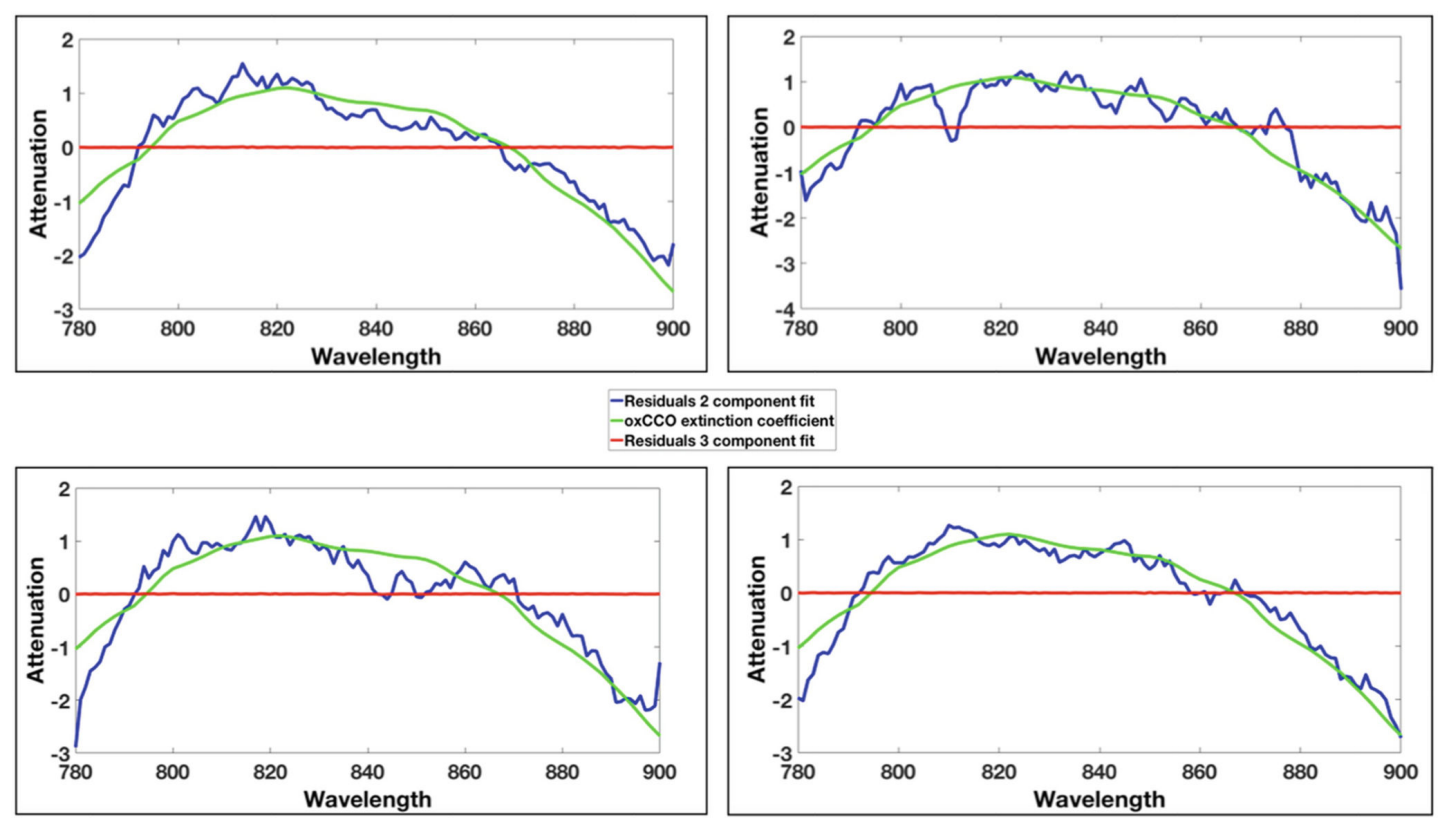
Residual analysis from 4 different participants

Figure 3 shows the results from the residual analysis for four different participants, chosen at random. The residual analysis indicates that the difference between the 3-component fit and 2-component fit is approximately the shape of the oxidized minus reduced CCO spectrum.

The one-sample Student’s t-test performed on the grand averaged data showed a significant increase from baseline in oxCCO with p_oxCCO_ = 0.000008, t_oxCCO_ = 5.710 with maximum concentration change in oxCCO being 0.238 ± 0.13 μM (p_HbO2_ = 0.000174, t_HbO2_ = 4.387, p_HHb_ = 0.382, t_HHb_ = −0.892, df = 23).

## 4 Conclusions

The residual analysis demonstrated that the difference between the 3-component fit and 2-component fit displays a broad peak around 830 nm and did not differ from the oxidized minus reduced CCO spectrum. This therefore suggests that changes that occur in the developing human brain during functional activation cannot be solely accounted for by Δ[HbO_2_] and Δ[HHb].

Our results demonstrate, for the first time in human infants, that cerebral changes in the oxidation state of CCO do occur during functional activation in infants and can be measured using non-invasive broadband spectroscopy to obtain a deeper understanding of cellular oxygen metabolism. A previous NIRS study reports diminished Δ[HbO_2_] responses to social stimuli in infants at-risk for autism [14, 15]. As CCO is a more direct marker of brain activation, CCO measurements alongside haemodynamics provide the opportunity to investigate physiological processes in the developing brain in a safe and non-invasive way and particularly to understand atypical brain development, such as by investigating the link between mitochondrial dysfunction and autism [16].
